# *Hanstruepera marina* sp. nov. and *Hanstruepera flava* sp. nov., two novel species in the family *Flavobacteriaceae* isolated by a modified *in situ* cultivation technique from marine sediment

**DOI:** 10.3389/fmicb.2022.957397

**Published:** 2022-07-22

**Authors:** Hong Ding, Jiahui Liu, Chen Yang, Chaobo Guo, Lijian Ding, Dawoon Jung, Weiyan Zhang

**Affiliations:** Li Dak Sum Yip Yio Chin Kenneth Li Marine Biopharmaceutical Research Center, College of Food and Pharmaceutical Sciences, Ningbo University, Ningbo, China

**Keywords:** in situ cultivation, modified ichip, *Hanstruepera*, polyphasic taxonomy, marine sediment

## Abstract

A modified *in situ* cultivation technique was developed and applied to resource mining of uncultured microbes from marine sediments of Meishan Island in the East China Sea. Two novel strains NBU2968^T^ and NBU2984^T^ were isolated by this method but not standard Petri dish, which indicated the modified technique was more effective compared to conventional approaches for isolating uncultured microbes and could be popularized and applied to other aquatic environments. The two novel strains were identified by the polyphasic taxonomic approach. Cells of both strains were observed to be Gram-staining-negative, rod-shaped, nonmotile, aerobic, and yellow-pigmented. Catalase and oxidase activities and hydrolysis of Tweens 40, 60, and 80 of two novel strains were positive. Methyl red reaction, H_2_S production, and hydrolysis of Tween 20 were negative. According to 16S rRNA gene sequence analysis, two novel strains shared the highest similarities (96.4–97.7%) to the species with a validated name in the genus *Hanstruepera*, while shared lower sequence similarities (<95.6%) to all other genera. Phylogenetic analysis revealed that strains NBU2968^T^ and NBU2984^T^ were affiliated with the genus *Hanstruepera*. ANI and dDDH values between the two novel strains and *Hanstruepera* species were 77.4–78.3% and 20.4–20.9%, respectively, which were below the thresholds for species delineation. The 16S rRNA gene sequence similarity, ANI, and dDDH values between the two novel strains were 99.3, 88.9, and 36.3%, respectively, indicating that the two strains represent different species. The genomes of NBU2968^T^ and NBU2984^T^ were 3.28 Mbp with a G+C content of 34.2% and 3.09 Mbp with a G+C content of 34.4%, respectively. The only respiratory quinone was menaquinone-6 (MK-6). The major cellular fatty acids were iso-C_15:0_, iso-C_15:1_G, and iso-C_17:0_ 3-OH. The major polar lipids of the two strains were phosphatidylethanolamine, unidentified amino lipids, and unidentified lipids. Based on the above polyphasic characteristics, strains NBU2968^T^ and NBU2984^T^ represent two novel species within the genus *Hanstruepera*, for which the names *Hanstruepera marina* sp. nov. and *Hanstruepera flava* sp. nov. are proposed. The type strains are NBU2968^T^ (= MCCC 1K06392^T^= KCTC 82913^T^) and NBU2984^T^ (= MCCC 1K07472^T^= KCTC 92511^T^), respectively.

## Introduction

The overwhelming majority of microbial species do not grow on synthetic media and remain unexplored (Zengler et al., 2002; Epstein, 2013; Jung et al., 2021a), which are known as “uncultivable” microorganisms. In recent years, novel cultivation methods have been advanced to access previously uncultivated microbes. A high-throughput *in situ* cultivation technique of ichip (isolation chip) was developed by Nichols et al. (2010), with highly efficient in terms of both microbial recovery and the novelty of isolated species. *In situ* cultivation techniques could better simulate natural conditions, and provide access to nutrients from the natural environment and critical growth factors supplied by neighboring species. Dormant microbes are able to stochastically wake into activity *in situ* if they detect suitable environmental factors or quorum sensing. Once the dormant cells become recovery, they tend to grow on artificial media (Buerger et al., 2012; Mu et al., 2018; Jung et al., 2021b). However, the original ichip device needs to use a gelling agent like agar to prepare a solid medium, it has two limitations: (1) the gelling agent like agar may inhibit the growth of part microbes or select to isolate some species; (2) the gelling agent act as a sieve, which can reduce the diffusion of nutrients and molecules. To overcome these shortcomings, we propose a modified *in situ* technique to combine the ichip and liquid dilution to extinction cultivation (Rappé et al., 2002; Yang et al., 2016; Oueriaghli et al., 2018) to isolate uncultured microorganisms. In this study, a modified ichip device was designed and applied to isolate uncultured microorganisms from marine sediments of Meishan Island in the East China Sea. Two novel strains NBU2968^T^ and NBU2984^T^ were isolated and studied, which belonged to the genus *Hanstruepera*.

The genus *Hanstruepera*, as a member of the family *Flavobacteriaceae*, was first reported by Hameed et al. (2015), to accommodate strictly aerobic, rod-shaped, nonmotile, and zeaxanthin-producing bacteria. It contained menaquinone-6 (MK-6) as the sole respiratory quinone and iso-C_15:0_, iso-C_15:1_ G, and iso-C_17:0_ 3-OH as predominant cellular fatty acids. At the time of writing, the genus *Hanstruepera* has only two recognized species (https://www.bacterio.net/genus/Hanstruepera:) *Hanstruepera neustonica* (type species), isolated from a surface water sample collected from an estuary (Hameed et al., 2015) and *Hanstruepera crassostreae*, isolated from an oyster sample collected from the coast (He et al., 2018). “*Hanstruepera ponticola*” renamed by Huang et al. (Huang et al., 2022), transferred from *Pseudobizionia ponticola* (Park et al., 2018) and isolated from seawater, was a heterotypic synonym of *Hanstruepera crassostreae* (Pei et al., 2021; Huang et al., 2022). In this article, we describe two novel strains NBU2968^T^ and NBU2984^T^ following the polyphasic taxonomic approach and propose that they represent two novel species of the genus *Hanstruepera*.

## Materials and Methods

### Design of the modified ichip device

**Figure 1:**
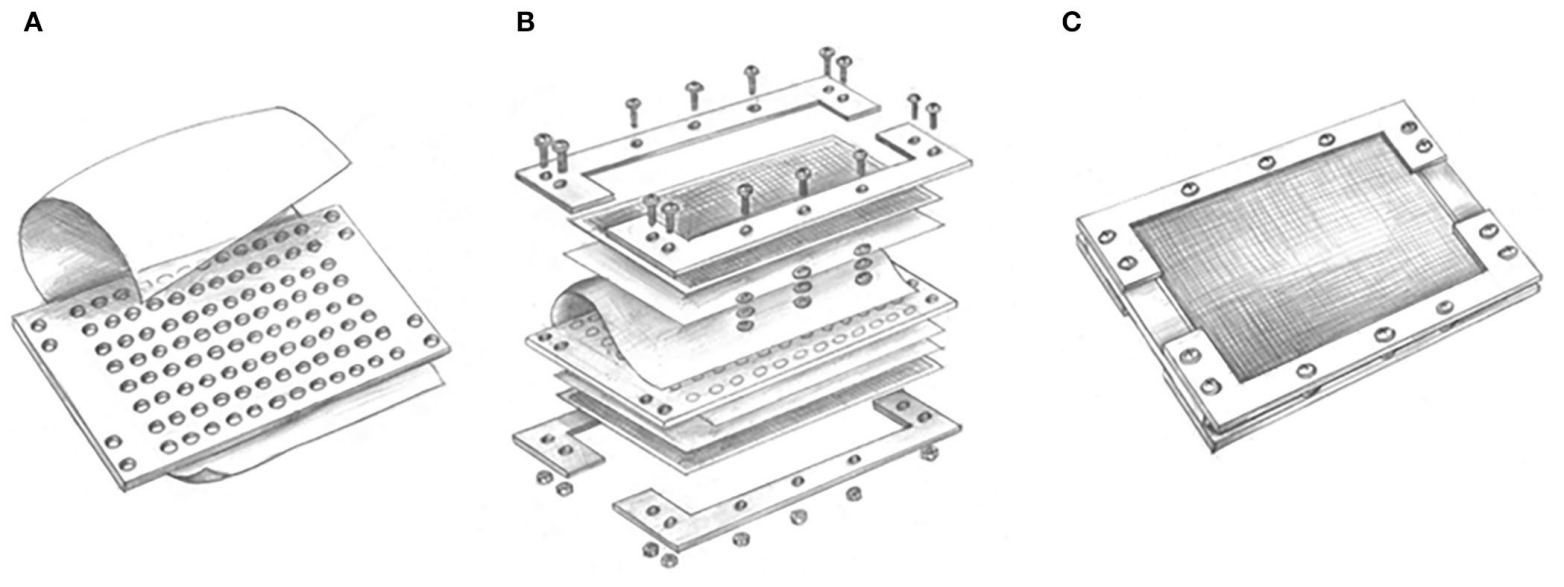
Conceptual design of modified ichip device: **(A)** A central plate including 96 through holes has added a suspension containing microbial cells in each hole and glued with the polycarbonate membranes by silicon glue; **(B)** nylon mesh and stainless steel mesh are, respectively, added on both sides of the plate, which are fixed with splints and screws; and **(C)** the assembled modified ichip device can be cultivated *in situ* environment.

The modified ichip device shares a similar concept and principle with the original one (Nichols et al., 2010; Berdy et al., 2017), which uses the dilution of bacteria of up to one to ten cells per well in microplates, but its incubation is performed in liquid media. The liquid medium potentially provides easier conditions for bacteria to adapt and promotes greater reproduction and growth compared to the solid medium containing the same nutrients. The modified ichip device consists of a central plate for cultivating bacterial cells, polycarbonate membranes with a 0.03 μm pore size on both the top and bottom of the plate, and side panels holding all other parts (Figure 1). Polytetrafluoroethylene (PTFE) is selected to be used as the material for the central plate, which is commercially produced, cheap and autoclavable. A total of 384 holes (in the original ichip device) are changed to 96 holes in the central plate, which correspond to wells of standard 96-well plates. The volume of holes increases from 1.25 to 50 μl, which prevents the medium inside the holes from drying out. Silicon glue is used to glue the polycarbonate membranes to each side of the central plate. It helps to ensure good sealing of the device and avoid microbial contamination from the outside or between holes. To protect polycarbonate membranes, nylon mesh and stainless steel mesh are, respectively, added on both sides of the plate, which are fixed with splints and screws. The assembled modified ichip device can be cultivated *in situ* environment (This method requires a substantial amount of moisture in the environment to prevent the liquid culture inside the ichip from drying out. Aquatic habitats are appropriate environments for the modified ichip application, but arid and semiarid systems are not.).

### Application of modified ichip device for isolation

Sediment samples were collected from the Meishan Island located in the East China Sea, Ningbo, China (121°56'E, 29°46'N) in December 2020. About 3.0 g sediment sample was vortexed and serially diluted to 1–10 cells/50 μl with 1/10 marine broth 2216 (MB). One polycarbonate membrane was glued to one side of the central plate, creating a 96-well plate with a membranous bottom. The cell suspension was aliquoted into the holes of modified ichip, and then the second membrane was glued to the top exposed part of the device. Leaving some compartments unoccupied and filled with sterile 1/10 MB as a control to detect the quality of the seal. Nylon mesh and stainless steel mesh helped to complete the assembly of the device. The assembled modified ichips were returned to the original sample environment for *in situ* cultivation, which helped the recovery and domestication of dormant microbes. After incubation of 4 weeks, the devices were retrieved to the lab. The modified ichips were washed with sterilized water to remove the microorganisms on the outer surfaces of the device. Sterile toothpicks were used to tear one side membrane, and the culture in the holes was transferred to 1/10 marine agar 2216 (MA). Colonies growing on 1/10 MA were picked and purified, and two novel isolates NBU2968^T^ and NBU2984^T^ were obtained. Standard Petri dish cultivation was used as control (about 3.0 g sediment sample was serially diluted to 10^−6^ with 1/10 MB, and 200 μl of each diluted sample was spread onto 1/10 MA plates), but these two novel strains were not isolated by this traditional method. These two strains were selected to be identified by the polyphasic taxonomic approach. *Hanstruepera crassostreae* MCCC 1H00246^T^ and *Hanstruepera neustonica* JCM 19743^T^ were selected as experimental control strains, which were obtained from the Marine Culture Collection of China (MCCC) and Japan Collection of Microorganisms (JCM), respectively. Both related type strains were cultured under the identical experimental conditions as strains NBU2968^T^ and NBU2984^T^ for comparative analysis.

### Phenotypic properties

Cell morphology was observed by using an optical microscope (BX40; Olympus) and transmission electron microscopy (JEM-1230; JEOL). Exponentially growing cells incubated on MA plates were suspended and stained with uranyl acetate and then fixed on the copper mesh before being observed with transmission electron microscopy. Gram staining was performed according to Dong and Cai (Dong and Cai, 2001). Motility was examined by microscopic observation and inoculation on semisolid MB medium with 0.5% agar (w/v). The presence of flexirubin-type pigments was investigated as described previously (Bernardet et al., 2002). To determine the growth conditions of strains NBU2968^T^ and NBU2984^T^, the temperature range for growth was determined in MB at 4, 10, 15, 20, 25, 28, 30, 35, 37, 40, 45, 50, and 55°C. The pH range for growth was determined at pH 4.0–10.0 (at intervals of 0.5) in MB supplemented with the following buffers: ammonium acetate (pH 4.0–5.0), MES (pH 5.5–6.0), PIPES (pH 6.5–7.0), Tricine (pH 7.5–8.5), and CAPSO (pH 9.0–10.0) at a concentration of 30 mM. The tolerance to NaCl was determined after cultivation at 32°C, pH 7.0 in modified MB medium with original Na^+^ and Cl^−^ removed (final NaCl concentration 0–10.0%, using increments of 1.0%, w/v). All tests of growth conditions were performed in quadruplicate and OD_600_ measurements were taken after 24-h incubation at 32°C with shaking at 140 rpm.

The following biochemical and physiological tests were carried out on strains NBU2968^T^, NBU2984^T^, *H. crassostreae* MCCC 1H00246^T^, and *H. neustonica* JCM 19743^T^ in MB, unless otherwise indicated. Catalase activity was detected *via* bubble production in a 3% (v/v) H_2_O_2_ solution. Oxidase activity was assessed by oxidation of 1% *p*-aminodimethylaniline oxalate. Indole production, methyl red, Voges–Proskauer test, H_2_S production, hydrolysis of starch, casein, and Tweens 20, 40, 60, and 80 were tested as described by Zhu et al. (2011). Other enzyme activities, physiological and biochemical properties, and acid production tests were determined by using API ZYM, API 20NE, and API 50CH strips (bioMérieux) according to the manufacturer's instructions. For the API 50CH test, we used modified MB in which yeast extract and peptone were replaced by 0.02 g/l yeast extract and 0.01 g/l phenol red. Anaerobic growth was determined with an AnaeroPack-MicroAero (2.5 l; MGC, Japan) anaerobic system by using sodium thiosulfate (20 mM), sodium sulfite (5 mM), sodium sulfate (20 mM), sodium nitrite (5 mM), or sodium nitrate (20 mM) as electron acceptors, respectively. Same media under aerobic condition were used as control. Susceptibility to antibiotics was investigated on MA using the disc diffusion method and considered susceptible when the diameter of the inhibition zone was over 1.2 cm (Sheu et al., 2020). The tested antibiotics were (μg per disc, unless indicated): amikacin (30), amoxicillin (20), ampicillin (10), bacitracin (0.04 IU), carbenicillin (100), cefamezin (30), cefoxitin (30), cefradine (30), cephalexin (30), chloramphenicol (30), ciprofloxacin (5), clindamycin (2), doxycycline (30), erythromycin (15), gentamicin (10), kanamycin (30), lincomycin (2), minocycline (30), nalidixic acid (30), neomycin (30), norfloxacin (10), novobiocin (30), nystatin (100), ofloxacin (5), oxacillin (1), penicillin G (10 IU), polymyxin B (300 IU), rifampicin (5), streptomycin (10), tetracycline (30), and vancomycin (30).

### Chemotaxonomic characteristic

Biomass for chemotaxonomic and molecular studies was obtained by cultivation in MB at 32°C for 24 h, with shaking at 140 rpm. All the following tests for chemotaxonomic characterization were performed on strains NBU2968^T^, NBU2984^T^, *H. crassostreae* MCCC 1H00246^T^, and *H. neustonica* JCM 19743^T^ unless otherwise indicated. For fatty acid methyl esters (FAMEs) analysis, late exponential-phase cells were harvested from MB. The identification and quantification of FAMEs were performed using the Sherlock Microbial Identification System (MIDI) with the standard MIS Library Generation Software version 6.1 according to the manufacturer's instructions. Respiratory quinones were extracted and analyzed by using reversed-phase HPLC as described by Minnikin et al. (1984). Total lipids were extracted as described by Kates (1986) and detected by two-dimensional TLC silica-gel 60 F_254_ aluminum-backed thin-layer plates (10 × 10 cm, Merck 5554), and further analyzed as described by Minnikin et al. (1984). The TLC plates were sprayed with phosphomolybdic acid with 5% ethanol to reveal total lipids and ninhydrin to reveal amino lipids (Zhang et al., 2015).

### Phylogeny analysis based on 16S rRNA gene sequences

The 16S rRNA gene was amplified by PCR using universal bacterial primers 27F (5'-AGAGTTTGATCCTGGCTCAG-3') and 1492R (5'-GGTTACCTTGTTACGACTT-3') (Sun et al., 2017). Purified PCR products were cloned into the vector pMD19-T (TaKaRa). The recombinant plasmid was transformed into *Escherichia coli* DH5α and then commercially sequenced. The almost-complete 16S rRNA gene sequences (1,487 nt) were compared with those of closely related species by EzBioCloud's Identify Service (http://www.ezbiocloud.net/identify) (Yoon et al., 2017a) and BLAST (https://blast.ncbi.nlm.nih.gov/Blast.cgi). Multiple sequence alignments and phylogenetic tree reconstructions were performed by using MEGA version 7.0 (Kumar et al., 2016). Phylogenetic trees were reconstructed by using three different methods: neighbor-joining (Saitou and Nei, 1987), maximum-likelihood (Felsenstein, 1981), and maximum-parsimony methods (Fitch, 1971). Evolutionary distances were calculated using the Kimura 2-parameter model (Kimura, 1980) for the neighbor-joining method. The topology of the phylogenetic trees was evaluated by using the bootstrap values based on 1,000 resamplings. *Crocinitomix catalasitica* NBRC 15977^T^ was selected as an outgroup.

### Genome sequencing and gene annotation

The whole genomes of strains NBU2968^T^and NBU2984^T^ were sequenced using an Illumina HiSeq 4000 system (Illumina) at the Beijing Genomics Institute (Shenzhen, China). The paired-end fragment libraries were sequenced according to the Illumina HiSeq 4000 system's protocol. Raw reads of low quality from paired-end sequencing (those with consecutive bases covered by fewer than five reads) were discarded. The sequenced reads were assembled using SOAPdenovo v1.05 software (Li et al., 2008). The open reading frames (ORFs) and the functional annotation of translated ORFs were predicted by using the RAST server online (Overbeek et al., 2014). The RNA genes were identified through tRNAscan-SE 2.0 (http://lowelab.ucsc.edu/tRNAscan-SE/, Lowe and Chan, 2016) and RNAmmer 1.2 server (http://www.cbs.dtu.dk/services/RNAmmer/, Lagesen et al., 2007). Metabolic pathways were analyzed by using the KEGG's BlastKOALA service (Kanehisa et al., 2016). Genome data publicly available of related *Hanstruepera* species were retrieved from the NCBI Genome database. The average nucleotide identity (ANI) values between strains NBU2968^T^, NBU2984^T^, and related species were calculated using the ANI calculator online service (Yoon et al., 2017b). Digital DNA-DNA hybridization (dDDH) values were calculated by the genome-to-genome distance calculator (GGDC) server version 2.1 (Meier-Kolthoff et al., 2013). Phylogenomic analysis was performed online by Type (strain) Genome Server (TYGS) (Meier-Kolthoff and Goeker, 2019).

## Results and discussion

### Phenotypic properties

Cells of strains NBU2968^T^ and NBU2984^T^ were Gram-negative, rod-shaped, and nonsporulating with no flagellum (Supplementary Figure 1). Colonies of two strains on MA incubated for 24 h were 1.0 mm in diameter, opaque, yellow-pigmented, and convex with a smooth surface. Flexirubin-type pigments are produced by two strains. Strain NBU2968^T^ grew at 0–6.0% (w/v) NaCl (optimum 2.0%, w/v), 10–40°C (optimum 37°C), and pH 5.5–8.0 (optimum pH 7.0), while strain NBU2984^T^ grew at NaCl 0–8.0% (w/v) (optimum, 2.0%), 15–37°C (optimum, 32°C), and pH 6.0–8.5 (optimum, pH 7.0) (Table 1). No growth occurred under the anaerobic condition on MA with the addition of different electron acceptors even after 2 weeks. Both strains were sensitive to amoxicillin, ampicillin, carbenicillin, cefamezin, cefoxitin, cefradine, cephalexin, chloramphenicol, ciprofloxacin, clindamycin, doxycycline, erythromycin, lincomycin, minocycline, norfloxacin, ofloxacin, penicillin G, rifampicin, streptomycin, and vancomycin. Strain NBU2968^T^ was sensitive to tetracycline but not for strain NBU2984^T^. Compared to two related type strains, strains NBU2968^T^ and NBU2984^T^ could grow without NaCl and hydrolyze Tween 80. Nitrate reduction was positive only for strain NBU2984^T^ (Table 1). Other physiological and biochemical characteristics are given in the species description (Table 1 and Supplementary Table 1).

**Table 1:** Differential characteristics of strain NBU2968^T^, strain NBU2984^T^, and related type strains of the genus *Hanstruepera*.

**Characteristic**	**1**	**2**	**3**	**4**
Habitat	Marine sediment	Marine sediment	Marine oyster^a^	Estuarine water^b^
Cell size (μm) Pigmentation	0.50–0.8 × 0.8–3.0 Yellow	0.4–0.6 × 1.0–2.4 Yellow	0.2–0.4 × 1.0–2.5^a^ Orange^a^	0.4–0.5 × 1.0–2.0^b^ Yellowish-orange^b^
Temperature range (optimum, °C)	10–40 (37)	15–37 (32)	4–40 (33)^a^	20–40 (30)^b^
pH range (optimum)	5.5–8.0 (7.0)	6.0–8.5 (7.0)	6.5–8.0 (7.5)^a^	6.0–8.0 (7.0)^b^
NaCl range (optimum) (%, w/v)	0–6.0 (2.0)	0–10.0 (2.0)	1.0–7.0 (3.0)^a^	2.0–4.0 (3.0)^b^
Voges-Proskauer Hydrolysis of: Casein Tween 20 Tween 80	+ - - +	- + - +	- + + -	- + + -
API 20NE test results:				
Fermentation of _D_-glucose Nitrate reduction, aesculin hydrolysis	+ -	+ +	+ -	- -
API ZYM test results:				
Trypsin, α-chymotrypsin *N*-acetyl-β-_D_-glucosaminidase, β-glucosidase Lipase (C14) Cystine arylamidase	- - + +	+ + - +	+ - - -	+ - - +
API 50CH test results:				
_D_-Glucose α-Methyl-_D_-mannopyranoside, α-methyl-_D_-glucopyranoside, amygdaline _L_-Arabinose, arbutin, _D_-galactose, gentiobiose, β-methyl-_D_-xylopyranoside, salicine, sucrose Aesculin, 5-ketogluconate Inositol, _D_-tagatose Cellobiose, _D_-xylose	+ + + - - +	+ - - + - -	+ + + - + -	- - + - + +
Susceptibility to				
Tetracycline Novobiocin Ciprofloxacin, streptomycin	S R S	R R S	R S R	S S S

*Taxa: 1, strain NBU2968^T^; 2, strain NBU2984^T^; 3, H. crassostreae MCCC 1H00246^T^; 4. H. neustonica JCM 19743^T^. All data were taken from this study unless otherwise indicated. Data marked with*

a *and*

b *were taken from He et al. (2018) and Hameed et al. (2015), respectively. -, negative; +, positive; R, resistant; S, susceptible. The same characteristics shared by these four strains were listed in Supplementary Table 1*.

### Chemotaxonomic properties

The predominant cellular fatty acids (>10%) of strain NBU2968^T^ were iso-C_15:1_ G (27.1%), iso-C_15:0_ (23.8%), and iso-C_17:0_ 3-OH (20.2%) and that of strain NBU2984^T^ consisted of iso-C_15:1_ G (25.0%), summed feature 3 (19.7%), iso-C_17:0_ 3-OH (17.5%), and iso-C_15:0_ (14.2%). The major fatty acids of two novel strains were similar to two related type strains (iso-C_15:1_ G, iso-C_15:0_, and iso-C_17:0_ 3-OH). The detailed fatty acid profile showed some differences among the four strains. For example, strain NBU2984^T^ possessed a higher amount of summed feature 3 but a lower amount of iso-C_15:0_ than the other three strains. Anteiso-C_15:1_ A was detected in strains NBU2968^T^ and NBU2984^T^, but not in two related type strains. Anteiso-C_15:0_, iso-C_16:0_, iso-C_16:1_ G, C_15:0_ 2-OH, and iso-C_16:0_ 3-OH were detected in strains NBU2968^T^, NBU2984^T^, and *H. neustonica* JCM19743^T^ but lacked in *H. crassostreae* MCCC 1H00246^T^, while C_15:1_ ω8*c* and summed feature 9 were only present in *H. crassostreae* MCCC 1H00246^T^ (Table 2). The only detected respiratory quinone in two novel strains was menaquinone-6 (MK-6), which was in common with the quinone type of the genus *Hanstruepera*. The major polar lipids were phosphatidylethanolamine (PE), unidentified amino lipids (ALs), and unidentified lipids (Ls), which were also in accordance with two related type species. The detailed polar lipid profile showed that strain NBU2984^T^ lacked L1, while the other three strains contained it. AL2 and AL3 were detected in two novel strains but not in two related type strains. AL5 was only present in *H. neustonica* JCM19743^T^ (Supplementary Figure 2).

**Table 2:** Cellular fatty acids for strains NBU2968^T^, NBU2984^T^, and related type strains of the genus *Hanstruepera*.

**Fatty acid**	**1**	**2**	**3**	**4**
**Saturated**				
C_10:0_	tr	-	2.4	-
C_16:0_	2.0	2.0	3.6	2.9
**Unsaturated**				
C_14:1_ ω5*c* C_15:1_ ω8*c*	tr -	tr -	1.3 2.0	tr -
**Branched** iso-C_14:0_ iso-C_15:0_ iso-C_15:1_ G anteiso-C_15:0_ anteiso-C_15:1_ A iso-C_16:0_ iso-C_16:1_ G **Hydroxy** C_15:0_ 2-OH iso-C_15:0_ 3-OH iso-C_16:0_ 3-OH iso-C_17:0_ 3-OH	1.0 **23.8** **27.1** 2.0 1.0 1.1 2.1 tr 5.0 1.5 **20.2**	1.1 **14.2** **25.0** 1.9 1.3 tr 1.2 2.3 6.0 2.0 17.5	1.3 **28.6** **25.4** - - - - - 5.7 - 20.2	tr **30.0** **28.4** tr - tr tr tr 4.8 tr 19.3
Summed feature 3^*^	9.4	19.7	6.7	6.3
Summed feature 9^*^	-	-	2.9	-

*Taxa: 1, strain NBU2968^T^; 2, strain NBU2984^T^; 3, H. crassostreae MCCC 1H00246^T^; 4, H. neustonica JCM19743^T^. All data were taken from this study. Values are percentages of the total fatty acid content. Fatty acids that represented <1% in all columns were omitted. Abbreviations: tr, trace component (<1%); -, not detected. Fatty acids present at >10% are indicated in bold*.

* *Summed feature 3 contained C_16:1_ ω7c and/or C_16:1_ ω6c; Summed feature 9 contained iso-C_17:1_ ω9c.and/or C_16:0_ 10-methyl*.

### Phylogeny of 16S rRNA gene sequences

**Figure 2:**
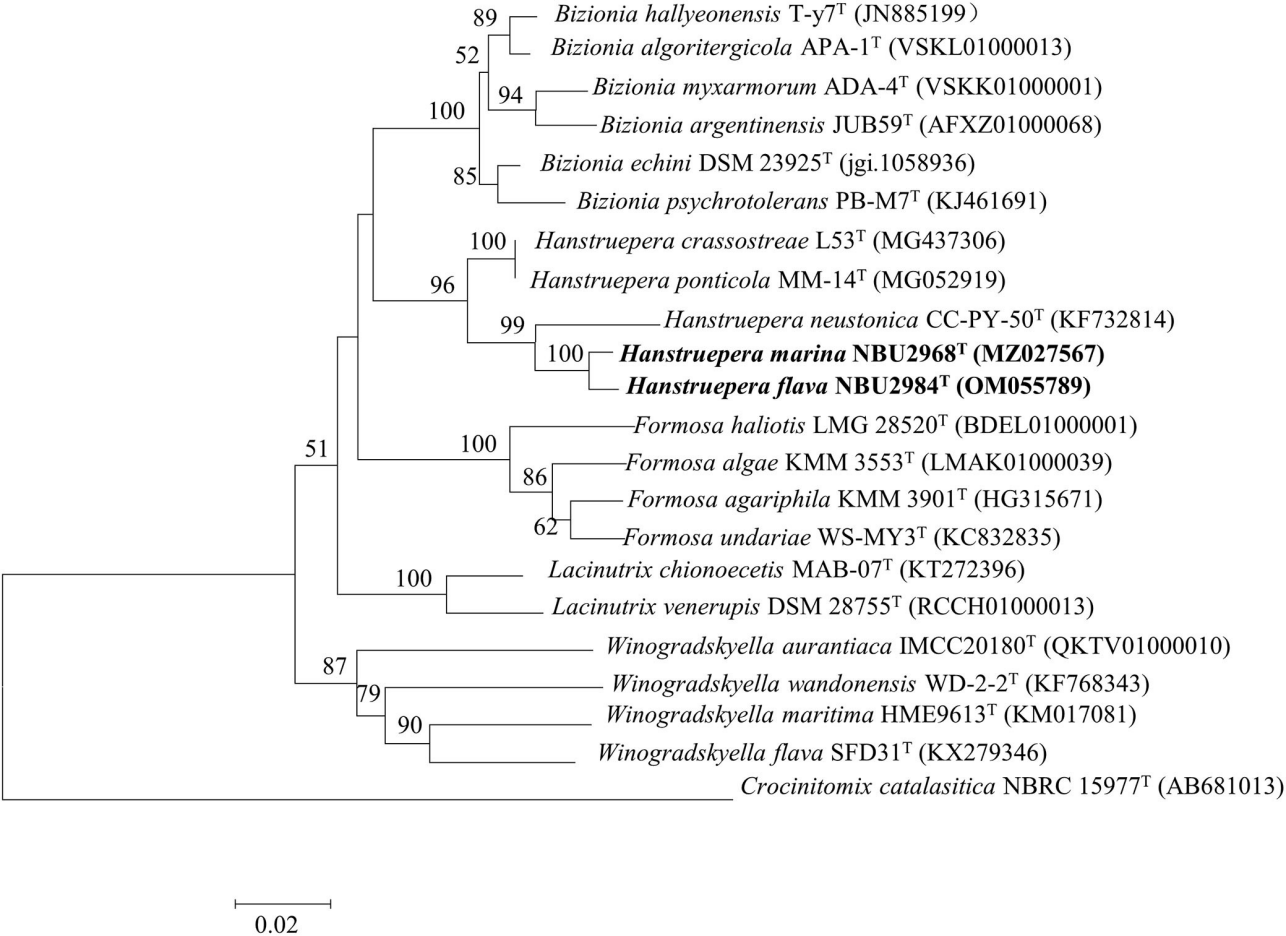
Neighbor-joining phylogenetic tree based on the 16S rRNA gene sequences, showing the phylogenetic relationships of strains NBU2968^T^, NBU2984^T^, and related taxa. Bootstrap values are based on 1,000 resamplings. Bootstrap values higher than 50% are indicated at branch points. Bar, 0.02 substitutions per nucleotide position.

The almost complete 16S rRNA gene sequences of strains NBU2968^T^ (1,487 bp, GenBank accession number: MZ027567) and NBU2984^T^ (1,487 bp, GenBank accession number: OM055789) were obtained through PCR amplification and sequencing. They shared 99.3% sequence similarity with each other. Sequence similarity searching in databases revealed that strains NBU2968^T^ and NBU2984^T^ shared the highest 16S rRNA gene sequence similarities (97.7%) with *H. crassostreae* L53^T^ and “*H. ponticola* MM-14^T^” (heterotypic synonym of *H. crassostreae* L53^T^), followed by *H. neustonica* CC-PY-50^T^ (96.4–96.6%), and shared low similarities (<95.6%) to other valid species. Phylogenetic analysis revealed that strains NBU2968^T^ and NBU2984^T^ were affiliated with species in the genus *Hanstruepera*, and closely related to *H. neustonica* CC-PY-50^T^, *H. crassostreae* L53^T^, and “*H. ponticola* MM-14^T^” on the different phylogenetic trees (Figure 2) and (Supplementary Figures 3, 4).

### Genomic characteristics

The draft genome sequence of strain NBU2968^T^ is composed of 13 contigs with the size of 3,282,034 bp, containing 3,065 protein-coding genes and 54 RNA genes. The draft genome sequence of strain NBU2984^T^ is composed of 23 contigs with the size of 3,094,910 bp, containing 2,880 protein-coding genes and 55 RNA genes. A total of 1,321 and 1,290 genes were assigned to KEGG for strains NBU2968^T^ and NBU2984^T^, respectively (Table 3). KEGG's analysis showed the major metabolic pathways of strains NBU2968^T^, NBU2984^T^, *H. crassostreae* L53^T^, and *H. neustonica* JCM19743^T^ were similar. They possessed the most genes in gene information processing, amino acid metabolism, and carbohydrate metabolism. All strains had complete pathways of gluconeogenesis (M00003), pyruvate oxidation (M00307), citrate cycle (M00009), pentose phosphate pathway (M00007), and phosphoribosyl diphosphate (PRPP) biosynthesis (M00005), whereas only strain NBU2984^T^ contained a complete glyoxylate cycle (M00012) (Table 4). In other metabolic pathways, the dTDP-L-rhamnose biosynthesis pathway (M00793) was found in all species. And a complete phosphatidylethanolamine (PE) biosynthesis pathway (M00093) was completely annotated in four strains, which was consistent with the polar lipids results of the genus *Hanstruepera*. In addition, the pathway of histidine biosynthesis (M00026) is complete in strains NBU2968^T^, NBU2984^T^, and *H. crassostreae* L53^T^ but not in strain *H. neustonica* JCM19743^T^, and only strain *H. crassostreae* L53^T^ did not possess complete C1-unit interconversion (M00140). Phylogenomic analysis showed that two novel strains were closely related to the genus *Hanstruepera* (Supplementary Figure 5), which was similar to NJ, ML, and MP trees. The genomic DNA G+C contents of NBU2968^T^ and NBU2984^T^ were 34.2 and 34.4%, respectively, which were close to two related type strains. The ANI and dDDH values between NBU2968^T^ and NBU2984^T^ were 88.9 and 36.3%, respectively, which were below the proposed species cut-off values of 95–96% for ANI and 70% for dDDH (Wayne et al., 1987; Goris et al., 2007), indicating that NBU2968^T^ and NBU2984^T^ represent two distinctive species. The ANI and dDDH values between the two strains and closely related *Hanstruepera* species were 77.4–78.3% and 20.4–20.9% (Table 3), respectively, indicating that the two strains represent novel species separated from validly published *Hanstruepera* species.

**Table 3:** The comparison of genomic features among strains NBU2968^T^, NBU2984^T^, and two species in the genus *Hanstruepera*.

**Features**	**1**	**2**	**3**	**4**
Genome size (bp)	3,282,034	3,094,910	3,136,223	3,049,585
Number of contigs	13	23	6	16
N50 length (bp)	569,330	1,585,466	2,516,937	499,047
G+C content (%)	34.2	34.4	33.5	35.4
Total Genes	3,119	2,935	2,931	2,842
Protein coding genes	3,065	2,880	2,875	2,802
RNA genes	54	55	39	40
Genes assigned to KEGG	1,321	1,290	1,269	1,265
GenBank accession no.	JAINVX000000000	JALJCW000000000	POTB01000000	POWF01000000
ANI/dDDH values (%, compared to NBU2968^T^)	/	88.9/36.3	77.9/20.8	77.5/20.5
ANI/dDDH values (%, compared to NBU2984^T^)	88.9/36.3	/	78.3/20.9	77.4/20.4

*Taxa: 1, strain NBU2968^T^; 2, strain NBU2984^T^; 3, H. crassostreae L53^T^; 4, H. neustonica JCM19743^T^*.

**Table 4:** The comparison of complete and incomplete metabolic pathways in the genomes of strains NBU2968^T^, NBU2984^T^, and related type strains of the genus *Hanstruepera*.

	**Pathway modules#**		**1**	**2**	**3**	**4**
Carbohydrate metabolism	Central carbohydrate metabolism	M00002 M00003 M00307 M00009 M00010 M00011 M00007 M00005	+ + + + + + + +	+ + + + + + + +	+ + + + + + + +	+ + + + + + + +
	Other carbohydrate metabolism	**M00012**	-	**+**	-	-
Lipid metabolism	Fatty acid metabolism	M00082 M00083 M00086	+ + +	+ + +	+ + +	+ + +
	Lipid metabolism	M00093	+	+	+	+
Nucleotide metabolism	Purine metabolism Pyrimidine metabolism	M00048 M00049 M00050 M00052 M00053	+ + + + +	+ + + + +	+ + + + +	+ + + + +
Amino acid metabolism	Serine and threonine metabolism Cysteine and methionine metabolism Lysine metabolism Histidine metabolism Aromatic amino acid metabolism	M00018 M00338 M00035 M00527 **M00026** M00045 M00023 M00038	+ + + + **+** + + +	+ + + + **+** + + +	+ + + + **+** + + +	+ + + + - + + +
Glycan metabolism	Lipopolysaccharide metabolism	M00063	+	+	+	+
Metabolism of cofactors and vitamins	Cofactor and vitamin metabolism	M00912 M00120 M00123 M00881 **M00140** M00121	+ + + + **+** +	+ + + + **+** +	+ + + + - +	+ + + + **+** +
Biosynthesis of terpenoids and polyketides	Terpenoid backbone biosynthesis Polyketide sugar unit biosynthesis	M00364 M00793	+ +	+ +	+ +	+ +

*Taxa: 1, strain NBU2968^T^; 2, strain NBU2984^T^; 3, H. crassostreae L53^T^; 4, H. neustonica JCM19743^T^. #: The description of pathway module numbers is listed in Supplementary Table 2. The different results of pathway module numbers shown in the table are indicated in bold*.

## Conclusion

A modified *in situ* technique was developed and applied to resource mining of uncultured microbes from marine sediments of Meishan Island in the East China Sea. Two novel strains NBU2968^T^ and NBU2984^T^ were isolated by this method but not standard Petri dish, which indicated the modified *in situ* technique was more effective for isolating uncultured microbes and could be popularized and applied to other aquatic environments. Based on the phenotypic, chemotaxonomic, phylogenetic data, and genome analysis, we conclude that strains NBU2968^T^ and NBU2984^T^ represent two novel species of the genus *Hanstruepera*, for which the names *Hanstruepera marina* sp. nov. and *Hanstruepera flava* sp. nov. are proposed, respectively.

### Description of *Hanstruepera marina* sp. nov.

*Hanstruepera marina* (ma.ri'na. L. fem. adj. *marina* of the sea, marine).

Cells are Gram-negative, aerobic, rod-shaped, and non-motile. The cell size is 0.5–0.8 × 0.8–3.0 μm. Colonies on Marine agar 2,216 are 1.0 mm in diameter, convex, smooth, opaque, and yellow-pigmented after 24 h at 32°C. Flexirubin-type pigments are present. The temperature range for growth is 10–40°C (optimum 37°C). Growth occurs at 0–6.0% NaCl and pH 5.5–8.0 (optimum, 2.0% NaCl and pH 7.0). Positive for catalase and oxidase activities, Voges–Proskauer, fermentation of _D_-glucose, arginine dihydrolase, hydrolysis of starch, gelatin, and Tweens 40, 60, and 80. Negative for methyl red, H_2_S production, indole production, β-galactosidase, urease, nitrate reduction, hydrolysis of casein, aesculin, and Tween 20. In the API ZYM kit, positive for activities of alkaline phosphatase, esterase (C4), esterase lipase (C8), lipase (C14), leucine arylamidase, valine arylamidase, cystine arylamidase, acid phosphohydrolase, and naphthol-AS-BI-phosphohydrolase. In the API 50CH kit, positive for _D_-glucose, glycogen, _D_-fructose, maltose, _D_-mannose, *N*-acetyl-β-_D_-glucosamine, lactose, 2-ketogluconate, starch, _D_-ribose, α-methyl-_D_-mannopyranoside, α-methyl-_D_-glucopyranoside, amygdaline, _L_-arabinose, arbutin, _D_-galactose, gentiobiose, β-methyl-_D_-xylopyranoside, salicin, sucrose, cellobiose, and _D_-xylose. The major fatty acids are iso-C_15:0_, iso-C_15:0_ G, and iso-C_17:0_ 3-OH. MK-6 is the only detected respiratory quinone. The polar lipids include phosphatidylethanolamine (PE), four unidentified amino lipids (ALs), and four unidentified lipids (Ls). The genomic DNA G+C content of the type strain is 34.2%.

The type strain NBU2968^T^ (= MCCC 1K06392^T^= KCTC 82913^T^) was isolated from a marine sediment sample taken from the Meishan Island in the East China Sea, China.

### Description of *Hanstruepera flava* sp. nov.

*Hanstruepera flava* (fla'va. L. fem. adj. flava yellow, the color of the pigment that the bacterium produces).

Cells are Gram-negative, aerobic, rod-shaped, and non-motile. The cell size is 0.4–0.6 × 1.0–2.8 μm. Colonies on MA are 1.0 mm in diameter, convex, smooth, opaque, and yellow-pigmented after 24 h at 32°C. Flexirubin-type pigments are present. The temperature range for growth is 15–37°C (optimum 32°C). Growth occurs at 0–10.0% NaCl and pH 6.0–8.5 (optimum, 2.0% NaCl and pH 7.0). Positive for catalase and oxidase activities, nitrate reduction, fermentation of _D_-glucose, hydrolysis of casein, starch, gelatin, aesculin, and Tweens 40, 60, and 80. Negative for methyl red, Voges–Proskauer, H_2_S production, indole production, arginine dihydrolase, β-galactosidase, urease, and hydrolysis of Tween 20. In the API ZYM kit, positive for activities of alkaline phosphatase, trypsin, α-chymotrypsin, *N*-acetyl-β-glucosaminidase, leucine arylamidase, esterase (C4), esterase lipase (C8), valine arylamidase, cystine arylamidase, acid phosphohydrolase, and naphthol-AS-BI-phosphohydrolase. In the API 50CH kit, positive for _D_-glucose, glycogen, aesculin, maltose, *N*-acetyl-β-_D_-glucosamine, _D_-fructose, lactose, _D_-ribose, starch, _D_-mannose, 2-ketogluconate, and 5-ketogluconate. The major fatty acids are iso-C_15:0_, iso-C_15:1_ G, iso-C_17:0_ 3-OH, and summed feature 3 (C_16:1_ ω7*c* and/or C_16:1_ ω6*c*). MK-6 is the only detected respiratory quinone. The polar lipids include phosphatidylethanolamine (PE), four unidentified amino lipids (ALs), and three unidentified lipids (Ls). The genomic DNA G+C content of the type strain is 34.4%. The type strain NBU2984^T^ (= MCCC 1K07472^T^= KCTC 92511^T^) was isolated from a marine sediment sample taken from the Meishan Island in the East China Sea, China.

## Data availability statement

The datasets presented in this study can be found in online repositories. The names of the repository/repositories and accession number(s) can be found in the article/supplementary material.

## Author contributions

WZ conceived the study. HD, JL, CY, and CG performed the experiments. WZ, LD, and DJ analyzed data. HD and CY wrote the manuscript. All authors read and approved the manuscript.

## Funding

This work was supported by the Natural Science Foundation of Zhejiang Province (LGF22C010001), the National Natural Science Foundation of China (32100001), the Ningbo Key Science and Technology Development Program (2021Z046), and Li Dak Sum Yip Yio Chin Kenneth Li Marine Biopharmaceutical Development Fund.

## Conflict of interest

The authors declare that the research was conducted in the absence of any commercial or financial relationships that could be construed as a potential conflict of interest.

## Publisher's note

All claims expressed in this article are solely those of the authors and do not necessarily represent those of their affiliated organizations, or those of the publisher, the editors and the reviewers. Any product that may be evaluated in this article, or claim that may be made by its manufacturer, is not guaranteed or endorsed by the publisher.

## Abbreviations

MA: marine agar 2216
MB: marine broth 2216
MCCC: Marine Culture Collection of China
KCTC: Korean Collection for Type Cultures
JCM: Japan Collection of Microorganisms
HPLC: high-performance liquid chromatography
MIDI: Microbial Identification System
TLC: thin layer chromatography
PE: phosphatidylethanolamine
AL: unidentified aminolipid
L: unidentified lipid
NCBI: National Center for Biotechnology Information
MEGA: Molecular Evolutionary Genetics Analysis
NJ: neighbor-joining
ML: maximum-likelihood
MP: maximum parsimony
RAST: Rapid Annotation using Subsystem Technology
KEGG: Kyoto Encyclopedia of Genes and Genomes
ANI: average nucleotide identity
dDDH: digital DNA-DNA hybridization.
