# Supplementary material for: Hanstruepera marina sp. nov. and Hanstruepera flava sp. nov., two novel species in the family Flavobacteriaceae isolated by a modified in situ cultivation technique from marine sediment
Source: Front Microbiol. 2022 Jul 22;13:957397. doi: 10.3389/fmicb.2022.957397 (PMC9355603; doi:10.3389/fmicb.2022.957397)
Supplement: Supplementary file 1 [file Presentation_1.pdf]

Supplementary Material

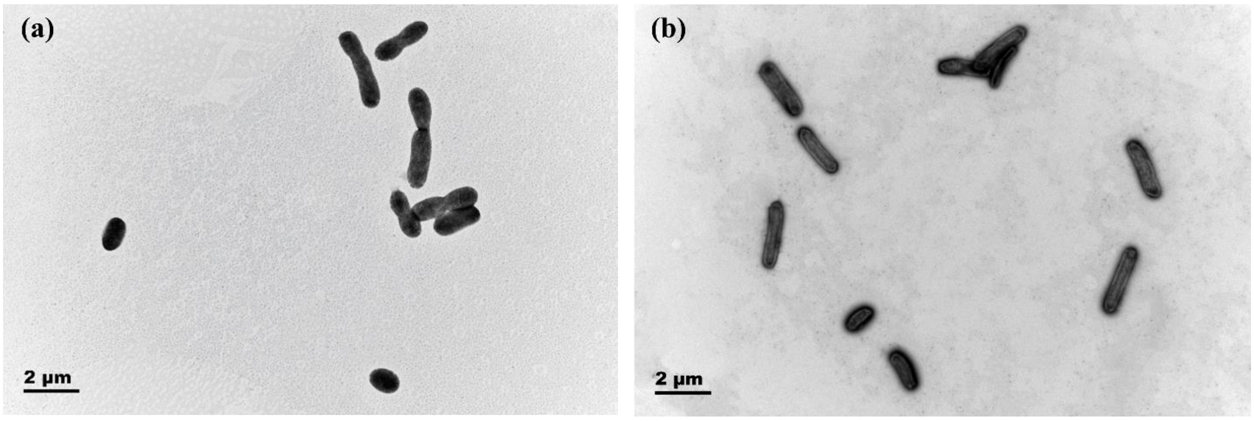

Supplementary Figure 1. Electron micrographs of cells of strains NBU2968^{T} (a) and NBU2984^{T} (b) growing on MA medium. Cells are rod-shaped with no flagellum. Bars, 2 μm.

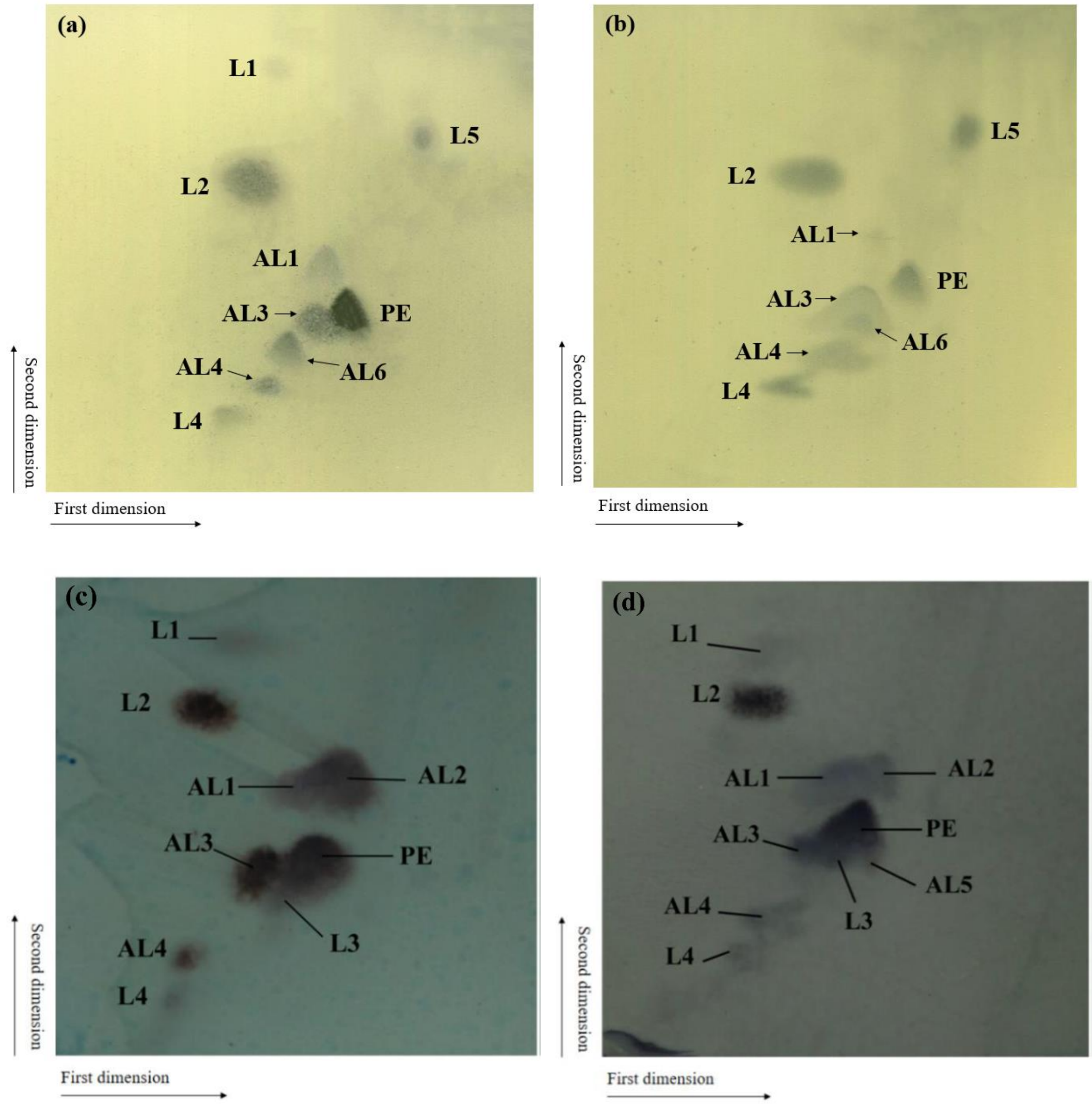

Supplementary Figure 2. Two-dimensional TLC plate images of polar lipids: (a) NBU2968^T, (b) NBU2984^T, (c) *H. crassostreae* L53^T and (d) *H. neustonica* JCM19743^T. Data of *H. crassostreae* L53^T and *H. neustonica* JCM19743^T were taken from He et al. (2018). First dimension (left to right) was developed with chloroform-methanol-water (65:25:4) and second dimension (bottom to up) was developed with chloroform-acetic acid-methanol-water (80:15:12:4). PE, phosphatidylethanolamine; AL, unidentified aminolipid; L, unidentified lipid. The following pictures were used phosphomolybdic acid (5 g phosphomolybdic acid hydrated in 100 ml ethanol) to reveal the total polar lipids.

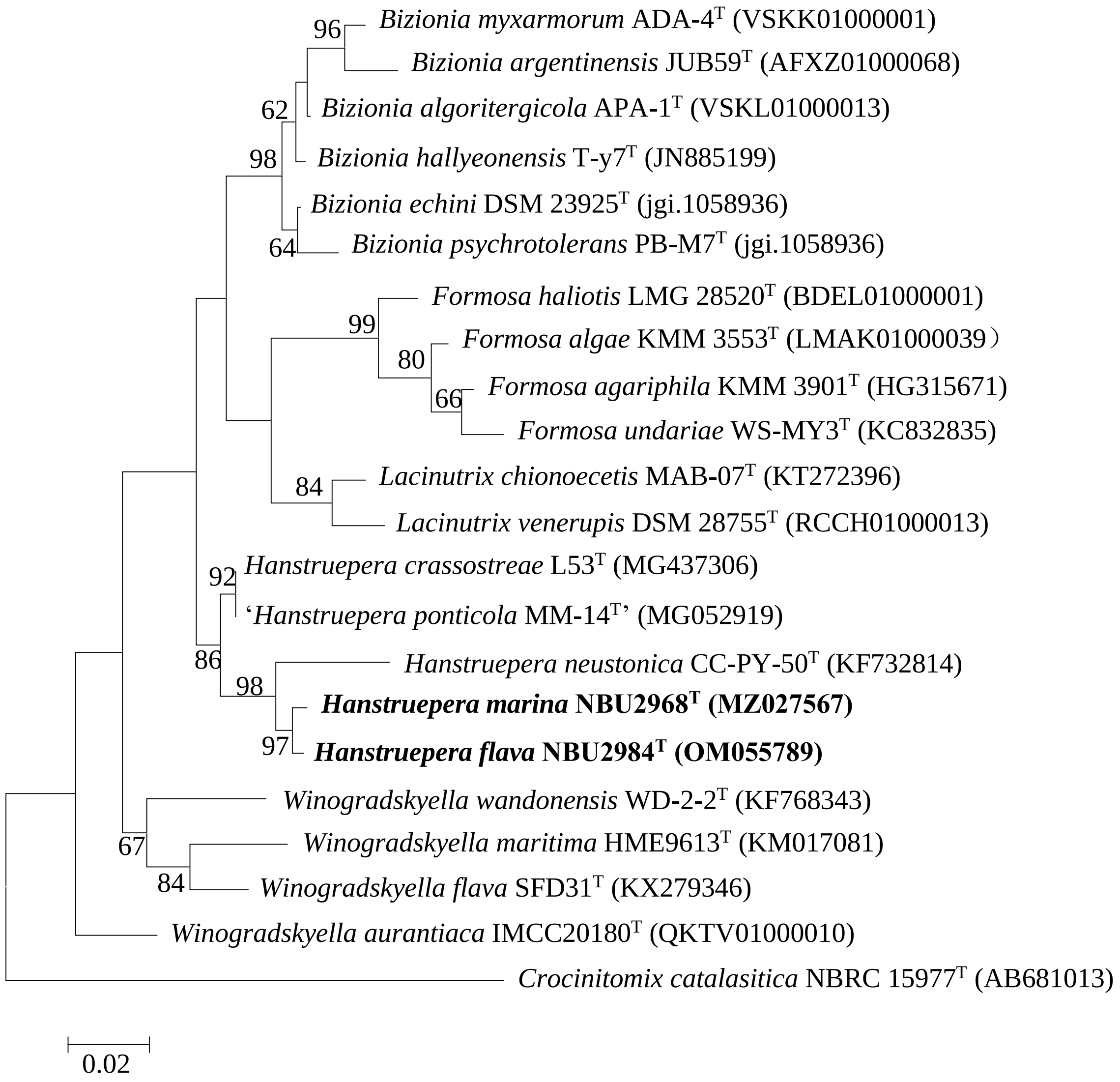

Supplementary Figure 3. Phylogenetic tree based on 16S rRNA gene sequences using maximum-likelihood method. Bootstrap values higher than 50% are indicated at branch-points. Bar, 0.02 substitutions per nucleotide position.

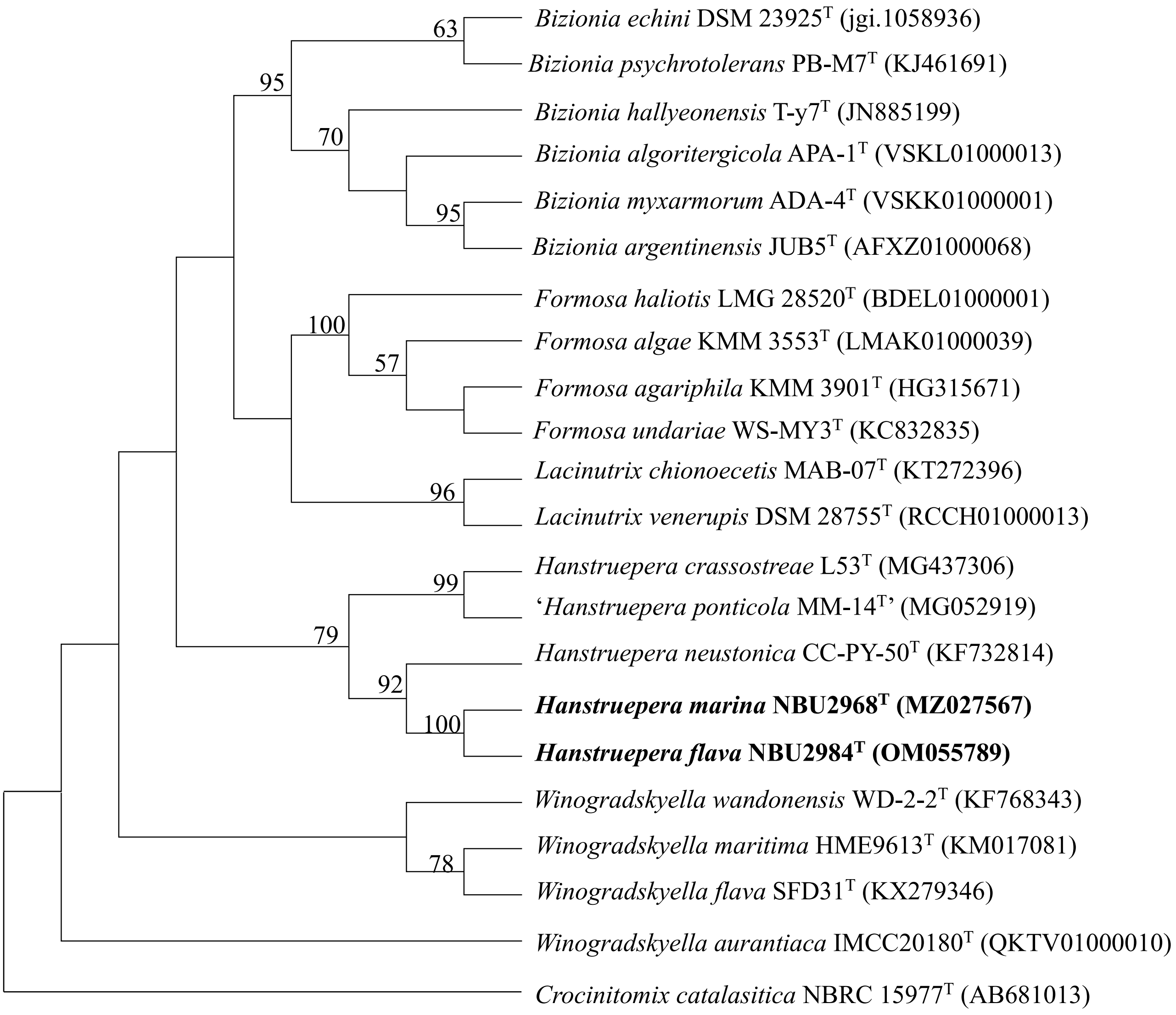

Supplementary Figure 4. Phylogenetic tree based on 16S rRNA gene sequences using the maximum-parsimony method. Bootstrap values higher than 50% are indicated at branch-points. Numbers at branching points refer to bootstrap values based on 1000 resamplings.

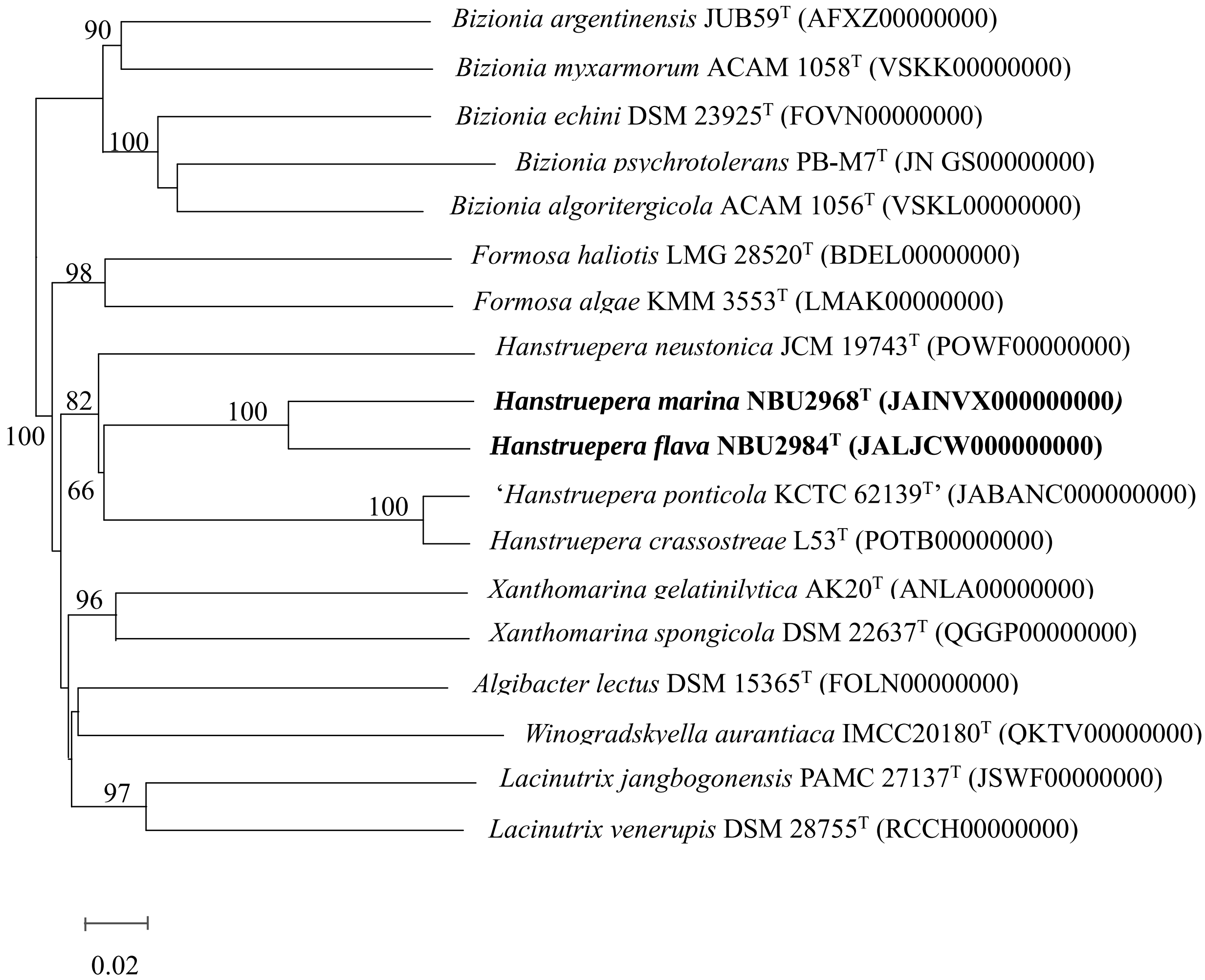

Supplementary Figure 5. Phylogenomic tree was generated by Type (strain) Genome Server (TYGS), showing the phylogenomic relationships of strains NBU2968^T, NBU2984^T and related taxa having genome sequences available.

Supplementary Table 1. The same characteristics of strains NBU2968^T, NBU2984^{T}and related type strains of the genus *Hanstruepera*.

Taxa: 1, strain NBU2968^T; 2, strain NBU2984^T; 3, *H. crassostreae* MCCC 1H00246^T; 4. *H. neustonica* JCM19743^T. All data were taken from this study unless otherwise indicated. Data marked with [a] and [b] were taken from He et al. (2018) and Hameed et al. (2015), respectively. -, negative; +, positive; R, resistant; S, susceptible.

Characteristic	1	2	3	4
Cell morphology	Rod	Rod	Rod[a]	Rod[b]
Oxygen requirement	Aerobic	Aerobic	Aerobic[a]	Aerobic[b]
Catalase and oxidase activities	+	+	+	+
H_2S production and Methyl red	-	-	-	-
Hydrolysis of starch, Tweens 40 and 60	+	+	+	+
API 20NE test results:				
Gelatin hydrolysis, arginine dihydrolase	+	+	+	+
Indole production, *β*-galactosidase, urease	-	-	-	-
API ZYM test results:				
Alkaline phosphatase, leucine arylamidase, valine arylamidase, acid phosphohydrolase, naphtol-AS-BI-phosphohydrolase, esterase (C4), esterase lipase (C8)	+	+	+	+
α-Fucosidase, *α*-galactosidase, *α*-glucuronidase, *α*-mannosidase, *β*-galactosidase, *α*-glucosidase	-	-	-	-
API 50CH test results:				
Glycogen, D-fructose, maltose, D-mannose, *N*-acetyl-*β*-D-glucosamine, lactose, 2-ketogluconate, starch, D-ribose	+	+	+	+
Glycerol, erythritol, D-arabinose, L-xylose, D-adonitol, D-sorbitol, D-xylitol, D-lyxose, L-fucose, D-arabitol, L-arabitol, D-gluconic acid, L-rhamnose, inositol, D-tagatose, dulcitol, D-mannitol, L-sorbose, D-fucose, inulin, melezitose, melibiose, raffinose, trehalose, D-turanose	-	-	-	-
Susceptibility to				
Ampicillin, penicillin G, lincomycin, clindamycin, doxycycline, erythromycin, ofloxacin, cephalexin, norfloxacin, chloramphenicol, cefoxitin, amoxicillin, rifampicin, cefamezin, carbenicillin, minocycline, cefradine, vancomycin	S	S	S	S
Nalidixic acid, kanamycin, neomycin, oxacillin, polymyxin B, bacitracin, nystatin, amikacin, gentamicin	R	R	R	R

Supplementary Table 2. The description of pathway modules numbers.

Pathway modules number	The description of metabolic pathways
M00002	Glycolysis, core module involving three-carbon compounds
M00003	Gluconeogenesis
M00307	Pyruvate oxidation
M00009	Citrate cycle (TCA cycle, Krebs cycle)
M00010	Citrate cycle, first carbon oxidation
M00011	Citrate cycle, second carbon oxidation
M00007	Pentose phosphate pathway, non-oxidative phase
M00005	PRPP biosynthesis
M00012	CAM (Crassulacean acid metabolism), dark
M00082	Fatty acid biosynthesis, initiation
M00083	Fatty acid biosynthesis, elongation
M00086	beta-Oxidation, acyl-CoA synthesis
M00093	Phosphatidylethanolamine (PE) biosynthesis
M00048	Inosine monophosphate biosynthesis
M00049	Adenine ribonucleotide biosynthesis
M00050	Guanine ribonucleotide biosynthesis
M00052	Pyrimidine ribonucleotide biosynthesis
M00053	Pyrimidine deoxyribonuleotide biosynthesis
M00018	Threonine biosynthesis
M00338	Cysteine biosynthesis
M00035	Methionine degradation
M00527	Lysine biosynthesis, DAP aminotransferase pathway
M00026	Histidine biosynthesis
M00045	Histidine degradation
M00023	Tryptophan biosynthesis
M00038	Tryptophan metabolism
M00063	CMP-KDO biosynthesis
M00912	NAD biosynthesis
M00120	Coenzyme A biosynthesis
M00123	Biotin biosynthesis
M00881	Lipoic acid biosynthesis, plants and bacteria
M00140	C1-unit interconversion, prokaryotes
M00121	Heme biosynthesis, plants and bacteria
M00364	C10-C20 isoprenoid biosynthesis, bacteria
M00793	dTDP-L-rhamnose biosynthesis
